# Supplementary material for: Image processing and Quality Control for the first 10,000 brain imaging datasets from UK Biobank
Source: Neuroimage. 2018 Feb 1;166:400–24. doi: 10.1016/j.neuroimage.2017.10.034 (PMC5770339; doi:10.1016/j.neuroimage.2017.10.034)
Supplement: Online data [file mmc1.pdf]

S1. Auto-shimming efficiency improvement

On 23/09/2014, a new auto-shimming approach was put in place with a reduced shimming field-of-view and fewer shim iterations. Some tests, described below, were performed in order to determine whether these changes resulted in any disruption to the data.

Shimming is the process of readjusting the homogeneity of a magnetic field during the scanning. In order to reduce the total acquisition time of the protocol[1], the number of shimming iterations was reduced to one, performed at the beginning of the acquisition protocol. Two features relating to the fMRI EPI data, "warping extent" and "drop-out" were checked and compared in 100 subjects from before the change and 100 after the change. "Warping extent" refers to the distortion correction displacement (distance), in voxels, represented as a displacement map estimated for fMRI images while "drop-out" refers to the intensity loss artefact (for example in temporal lobes) in fMRI EPI images. It is worth noting that we were not looking for these values to improve, but rather to achieve similar levels of warping/dropout despite the reduced time spent on shimming.

- "Warping extent": Figure S1.A shows that the distortion correction estimated for EPI images was not substantially affected by the shimming change (the histograms show a very slight reduction in distortion with the latter shimming method).
- "Drop-out": We defined dropout regions as being brain regions where intensity (averaged across subjects) falls to less than 20% of the global mean intensity. Figure S1 (panels B and C) shows the mean signal drop-out (focused on the temporal lobes) for 100 subjects before and after the shimming change, with only minor differences apparent. There were no significant changes in mean intensity in dropout regions after correcting for multiple comparisons across space. At a more liberal testing threshold ($P < 0.01$, two-tailed t-test, uncorrected for multiple comparisons), there was a small medio-frontal area of change in signal level ($t_{200} = 3.4$); this showed less dropout with the latter (customised) shimming approach as can be seen in Figure S1.D. This is also confirmed with histograms in Figure S1.E, which shows strong similarity of the intensity distributions in rfMRI before and after the change.

The primary reason that the reduction in EPI warping and drop-out do not get worse here with fewer shimming iterations, is that the volume the shim is optimised over is now reduced and (accordingly) the shim data resolution is improved.

[1] Time saved with this change was 1 minute 28 seconds.

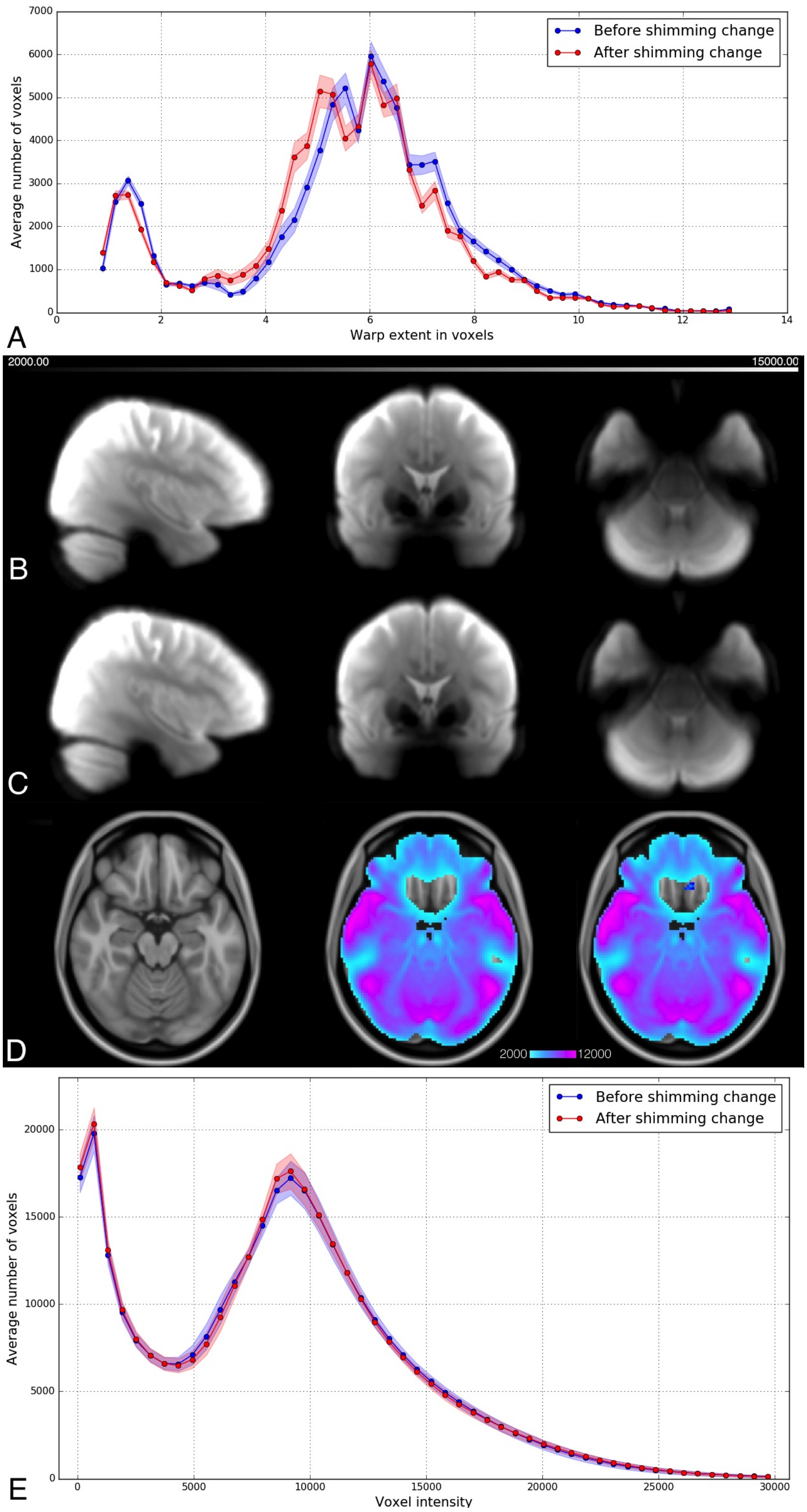

Figure S1: Tests to verify that the change in shimming did not affect the data significantly. *A:* Histogram across voxels of the undistortion warp extent before and after the shimming change (Ribbon = standard deviation) averaged across subjects. *B:* Mean drop-out for 100 subjects before shimming change and (C) after shimming change. *D left:* MNI152 T1 template. *D middle:* Mean fMRI, original shimming thresholded at 20% global mean. *D right:* Mean fMRI, customised shimming thresholded at 20% global mean. Difference between two shimming methods in dark blue (P <0.01 uncorrected). *E:* Histogram across voxels of the mean intensity of EPI images across 100 subjects (Average of rfMRI) (Ribbon = standard deviation).

S2. Algorithmic rules for modality selection

Algorithm 1 Logic to manage T1w images

```
Require: T1 images, T1 directory, unclassified directory, raw directory. {Same rules apply to T2 FLAIR.}
if (num(T1) >= 2) then
  if (num(T1) = 2) then
    Normalised T1 ⇒ T1 directory {Check JSON file}
    Not normalised T1 ⇒ raw directory {Check JSON file}
  else
    Last Normalised T1 ⇒ T1 directory {In acquisition order}
    Last not Normalised T1 ⇒ raw directory {In acquisition order}
    Rest of T1s ⇒ unclassIFied directory {IF any}
  end if
end if
```

Algorithm 2 Logic to manage fMRI images

```
Require: fMRI images, fMRI directory, unclassified directory,. {Same rules apply to rfMRI and tfMRI.}
if (num(fMRI images) = 1) then
  if (fMRI image is 4D) then
    generate SBRef(fMRI image) {The image did not have SBRef}
    fMRI image + SBRef ⇒ fMRI directory
  end if
else if (num(fMRI images) = 2) then
  BiggestImage ⇐ max(dim(fMRI images)) {Find the biggest image}
  if BiggestImage is 4D then
    if SmallestImage is 3D then
      SBRef ⇐ SmallestImage
    else
      generate SBRef(BiggestImage) {The image did not have SBRef}
    end if
    fMRI image + SBRef ⇒ fMRI directory
    Rest of fMRIs ⇒ unclassIFied directory {If any}
  end if
else
  BiggestImage ⇐ max(dim(fMRI images)) {Find the biggest image}
  if BiggestImage is 4D then
    if Previous to BiggestImage is 3D then
      SBRef ⇐ Previous to BiggestImage
    else
      generate SBRef(BiggestImage) {The image did not have SBRef}
    end if
    fMRI image + SBRef ⇒ fMRI directory
    Rest of fMRIs ⇒ unclassIFied directory {If any}
  end if
end if
```

Algorithm 3 Logic to manage dMRI images

Require: dMRI images, dMRI directory, unclassified directory
  for all dMRI *in* dMRI images **do**
    if EncodingDirection(dMRI) = AP **then**
      dMRI ⇒ APs
    else
      dMRI ⇒ PAs
    end if
  end for
  for group *in* (APs, PAs) **do**
    if (num(group) ≥ 1) **then**
      BiggestImage ⇐ max(dim(group)) {Find the biggest image}
      BiggestImage + bval + bvec ⇒ dMRI directory
      Rest of dMRIs *in* group ⇒ unclassified directory {If any}
    end if
  end for

Algorithm 4 Logic to manage swMRI images

```
Require: swMRI images, PHASE TE1 directory, PHASE TE2 directory
Require: MAGNITUDE TE1 directory, MAGNITUDE TE2 directory
Require: swMRI directory, swMRI unclassIFied directory
  if (num(swMRI images) = 134 then
    for all swMRI in swMRIimages do
      if swMRI is coil image then
        swMRI ⇒ coilImages
      else
        swMRI ⇒ mainImages
      end if
    end for
    for all swMRI in coilImages do
      if swMRI is phase image then
        if TE(swMRI) = TE1 then
          swMRI ⇒ PHASE TE1 directory
        else
          swMRI ⇒ PHASE TE2 directory
        end if
      else
        if TE(swMRI) = TE1 then
          swMRI ⇒ MAGNITUDE TE1 directory
        else
          swMRI ⇒ MAGNITUDE TE2 directory
        end if
      end if
    end for{Finish with Coil Images}
    if num(swMRI Phase TE1) in mainImages = 1 then
      swMRI Phase TE1 ⇒ swMRI directory
    else if num(swMRI Magnitude TE1) in mainImages = 2 then
      swMRI Magnitude TE1 ⇒ swMRI directory
    else if num(swMRI Phase TE2) in mainImages = 1 then
      swMRI Phase TE2 ⇒ swMRI directory
    else if num(swMRI Magnitude TE2) in mainImages = 2 then
      swMRI Magnitude TE2 ⇒ swMRI directory
    end if{Finish with Main Images}
    for all swMRI not classified do
      swMRI ⇒ swMRI unclassified directory
    end for
  end if
```

S3. IDP list

There are 4353 publicly available IDPs[2] in the UK Biobank showcase[3]. Here we summarise them:

- 3 IDPs: Discrepancy between the T1 structural image and the standard (population average) template image (See Section S4).
- 2 IDPs: Reciprocal of the signal to noise ratio (SNR) and contrast to noise ratio (CNR) of the T1 (See Section S4).
- 5 IDPs: Cost function for the linear alignment of the 5 modalities to the T1.
- 2 IDPs: Head motion for resting-state fMRI and task fMRI.
- 3 IDPs: Reciprocal of the temporal signal to noise ratio (TSNR) for resting-state fMRI, cleaned resting-state fMRI (After running FIX) and task fMRI.
- 1 IDP: Number of dMRI outlier slices (across all slices in all volumes) detected and corrected with eddy.
- 11 IDPs: SIENAX outputs for normalised and non-normalised volumes (See Section S4).
- 15 IDPs: Volumes of the 15 subcortical structures identified with FIRST (See Section S4).
- 139 IDPs: Volumes in grey matter in 139 cortical structures.
- 1 IDP: Total volume of white matter hyperintensities using BIANCA.
- 14 IDPs: Median T2* intensity (from SWI data) in 7 paired subcortical structures from FIRST.
- 12 IDPs: Median and 90th percentile of the 3 main contrasts in the task fMRI analysis (shapes, faces and faces-shapes) and their corresponding zstats.
- 4 IDPs: Median and 90th percentile of the faces-shapes contrast in the task fMRI analysis in the amygdala and their corresponding zstats.
- 432 IDPs: Mean intensity for dtifit outputs (FA, MD, MO, L1, L2, L3) and NODDI outputs (ICVF, OD, ISOVF) for the 48 tracts segmented with the TBSS-like analysis.
- 243 IDPs: Mean intensity for dtifit outputs (FA, MD, MO, L1, L2, L3) and NODDI outputs (ICVF, OD, ISOVF) for the 27 tracts segmented with the probabilistic tractography analysis.
- 21 IDPs: Amplitudes for the 21 nodes analysed in the functional network connectivity analysis with low dimensionality (25 components before clean-up).
- 210 IDPs: Full normalised temporal correlation between every node for the 21 nodes analysed in the functional network connectivity analysis with low dimensionality (25 components before clean-up).
- 210 IDPs: Partial temporal correlation between every node for the 21 nodes analysed in the functional network connectivity analysis with low dimensionality (25 components before clean-up).
- 55 IDPs: Amplitudes for the 55 nodes analysed in the functional network connectivity analysis with high dimensionality (100 components before clean-up).
- 1485 IDPs: Full normalised temporal correlation between every node for the 55 nodes analysed in the functional network connectivity analysis with high dimensionality (100 components before clean-up).
- 1485 IDPs: Partial temporal correlation between every node for the 55 nodes analysed in the functional network connectivity analysis with high dimensionality (100 components before clean-up).

[2] http://www.fmrib.ox.ac.uk/datasets/ukbiobank/nnpaper/IDPinfo.txt
[3] http://biobank.ctsu.ox.ac.uk/crystal/refer.cgi?id=9028 .

S4. QC features

Some of the QC features presented here (the first 31) are also IDPs for T1 images as listed above. We have divided these features into 17 groups:

- The first three features (QC_T1_001 to QC_T1_003) describe the discrepancy between the T1 structural image and the standard (population average) template image. This discrepancy (generalised difference) implies that an extreme score could arise either because the quality of the alignment is bad, or because one of the two images being compared is different in some way, either due to artefacts or biological differences. The 3 metrics are: the discrepancy after linear alignment (which cannot correct for fine-grained differences between a given subject and the population average); the discrepancy after non-linear alignment (which should achieve much better correspondence); and the overall amount of nonlinear warping (utilising the Jacobian of the non-linear warp field) necessary to achieve a detailed alignment to the standard template.
- The next two features (QC_T1_004 and QC_T1_005) measure signal to noise ratio (SNR) of the T1. Brain and tissue-type segmentation[4] is used to estimate background noise level (standard deviation), as well as mean intensities for grey and white matter. These quantities are used to estimate overall image SNR and also CNR (contrast to noise - white-grey mean intensity difference normalised by noise level). In both cases these measures are inverted before being recorded as QC measures, so that higher is worse. As with the previous features, similar measures can be calculated from preprocessed rfMRI (both before and after artefact removal) and tfMRI timeseries data. In this case however the "noise" level is the temporal standard deviation.
- The next eleven features (QC_T1_006 to QC_T1_016) show SIENAX outputs (see Section 2.2.2 of the paper) with the normalised and non-normalised volumes for the whole brain, and for the segmented tissue types. These metrics can be useful in identifying problems in the T1 acquisition as well as the registration and brain extraction, as the segmentation strongly depends on the brain extraction.
- The next fifteen features (QC_T1_017 to QC_T1_031) show the volumes of the 15 subcortical structures identified in the T1[5]. Extreme cases of these features can come either from acquisition or processing problems, or from variability that may be worth studying (and therefore may not represent a problem in the processing, but rather anatomical variability).
- The next seven features (QC_T1_032 to QC_T1_038) show the asymmetry defined as the ratio of larger volume / smaller volume between the 7 paired subcortical structures from the previous block.
- The next eight features (QC_T1_039 to QC_T1_046) show the global asymmetry in the brain calculated as the difference between the brain-extracted T1 and the same image flipped in the left-right axis (after a prior linear registration to the MNI152 template and normalisation with the median intensity of the T1). The 8 metrics cover various descriptive statistics calculated from this difference (such as maximum value, mean value, etc.). A very strong global asymmetry can be due to extreme structural variability (which is one of the cases described in Section 2.4.1 of the paper) or problems in acquisition or processing.
- The next sixty features (QC_T1_047 to QC_T1_106) contain the mean and standard deviation of the 15 main subcortical structures normalised using 2 different methods:

[4] Output of the FAST tool.
[5] Output of the FIRST tool.

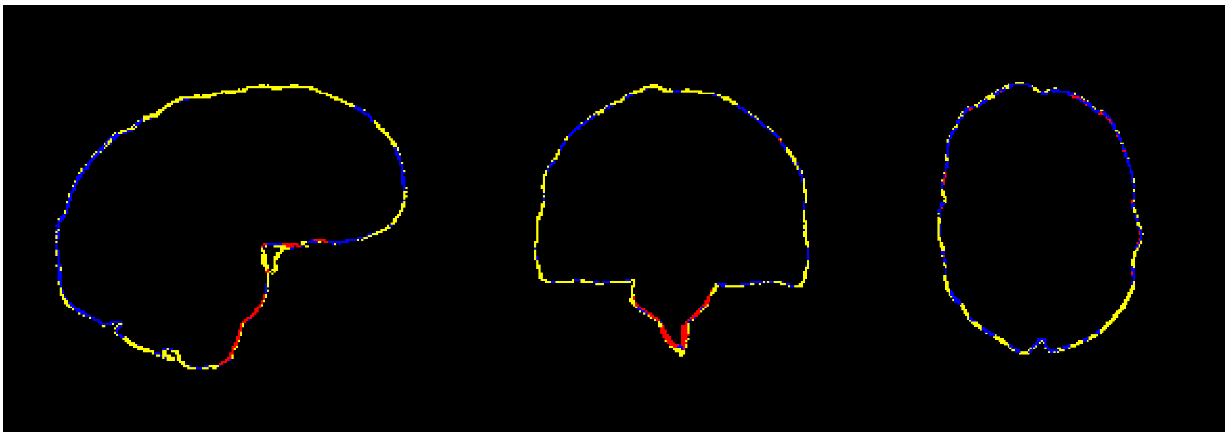

Figure S2: Volume of different tissues on the edges of the standard brain mask. *Red:* White matter (2,389 mm^3). *Blue:* Grey matter (44,696 mm^3). *Yellow:* CSF (62335 mm^3).

  - Method 1: Subtracting the mean intensity in the grey matter from the mean intensity in the structure and dividing by the difference between mean intensity in white matter and mean intensity in grey matter.
  - Method 2: Dividing mean intensity in the structure by mean intensity in grey matter.

- The next feature (QC_T1_107) contains the volume of grey matter that lies outside of the subject's brain mask. This grey matter volume was calculated with FAST on a brain extracted T1 using BET (As opposed to the brain extraction method described in Section 2.2.2 of the paper). The idea behind this feature would be to be able to detect grey matter out of the brain mask calculated by our pipeline using an alternative brain extraction method.

- The next nine features (QC_T1_108 to QC_T1_116) contain the amount of all tissues from the FAST segmentation that lie on the edges of the subject's brain mask. An example of this can be seen in Figure S2. These features could be useful for identifying problems in brain extraction and registration, as the amount of white matter should be negligible on the edges of the brain (the edges should primarily consist of grey matter / CSF).

- The next seven features (QC_T1_117 to QC_T1_123) contain the volume of edges calculated with a Canny filter on the brain extracted T1. Edges that lie on the brain mask boundary, the ventricles and the brainstem are excluded. An example is shown in Figure S3. The utility of these features is very similar to the tissue edge features described above.

- The next eleven features (QC_T1_124 to QC_T1_134) describe a comparison (using the Dice coefficient in a similar manner to Kazemi and Noorizadeh (2014)) between the Biobank brain mask (as described in section 2.2.2 of the paper) and other brain extraction methods, including:
  - Non linear registration to a study specific template (As opposed to the MNI152 template).
  - mriwatershed method from Freesurfer (Ségonne et al., 2004) using default parameters.
  - mriwatershed method from Freesurfer using the atlas option.
  - ROBEX (Iglesias et al., 2011).
  - SPM (Ashburner and Friston, 2005).
  - BSE from BrainSuite (Shattuck and Leahy, 2002) with default paramenters.

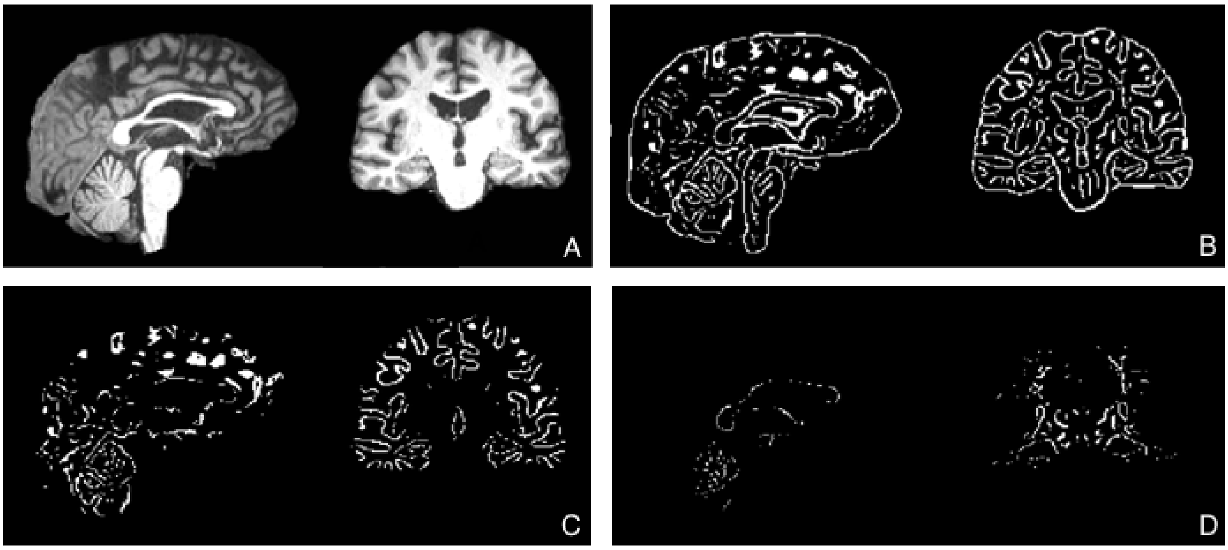

Figure S3: Volume of edges identified using a Canny filter on different tissue types. *A:* Brain extracted T1. *B:* Edges in the whole brain. *C:* Edges in the GM using FAST segmentation and cleaning edges on the brain mask boundary, ventricles and brain stem. *D:* Edges in the WM using the same cleaning as in C.

- BSE from BrainSuite with optimised paramenters[6].
- BET (Smith, 2002) on T1 image.
- BET (Smith, 2002) on T2 FLAIR image.
- Linear registration of the MNI brain mask to T1 space.
- Mask created by selecting the voxels that belong to a majority (Number of available masks −2) of the previously created masks.

With these features, we are attempting to avoid the circularity of evaluating the quality of Biobank brain extraction by using features which depend upon that brain extraction.

- The next six features (QC_T1_135 to QC_T1_140) contain 6 descriptive statistics (mean, median, and 2nd, 25th, 75th, and 98th percentile) of the 11 previously mentioned DICE coefficients for the brain extraction methods described above.

- The next twelve features (QC_T1_141 to QC_T1_152) contain 3 descriptive statistics (mean, median and 95th percentile) of the intensity of the internal and external boundary of the brain mask. We calculated the internal and external boundaries on the whole brain mask and on the upper half of the brain mask (As that is the portion of the brain with the larger number of problems in the registration). See Figure S4.

- The next two features (QC_T1_153 and QC_T1_154) contain the volume of the largest hole in the grey matter and white matter (With a "hole" defined as a cluster of voxels with intensity below the 5th percentile). The largest hole is calculated using a Z threshold of 0.5.

- The next five features (QC_T1_155 to QC_T1_159) contain some descriptive statistics (Mean, median, 75th and 99th percentile, and number of voxels with extreme values[7]) of the magnitude of the non-linear registration to the MNI152 template calculated as the square root of the sum of the three squared dimensions of the estimated warp field.

[6]Diffusion constant = 50; Diffusion iterations = 1 ; Edge detection constant = 0.7 (-d 50 -n 1 -s 0.7). These parameters were optimised with a grid search, using similarity with Biobank brain mask as the cost function.

[7]Mean + 3 times the standard deviation.

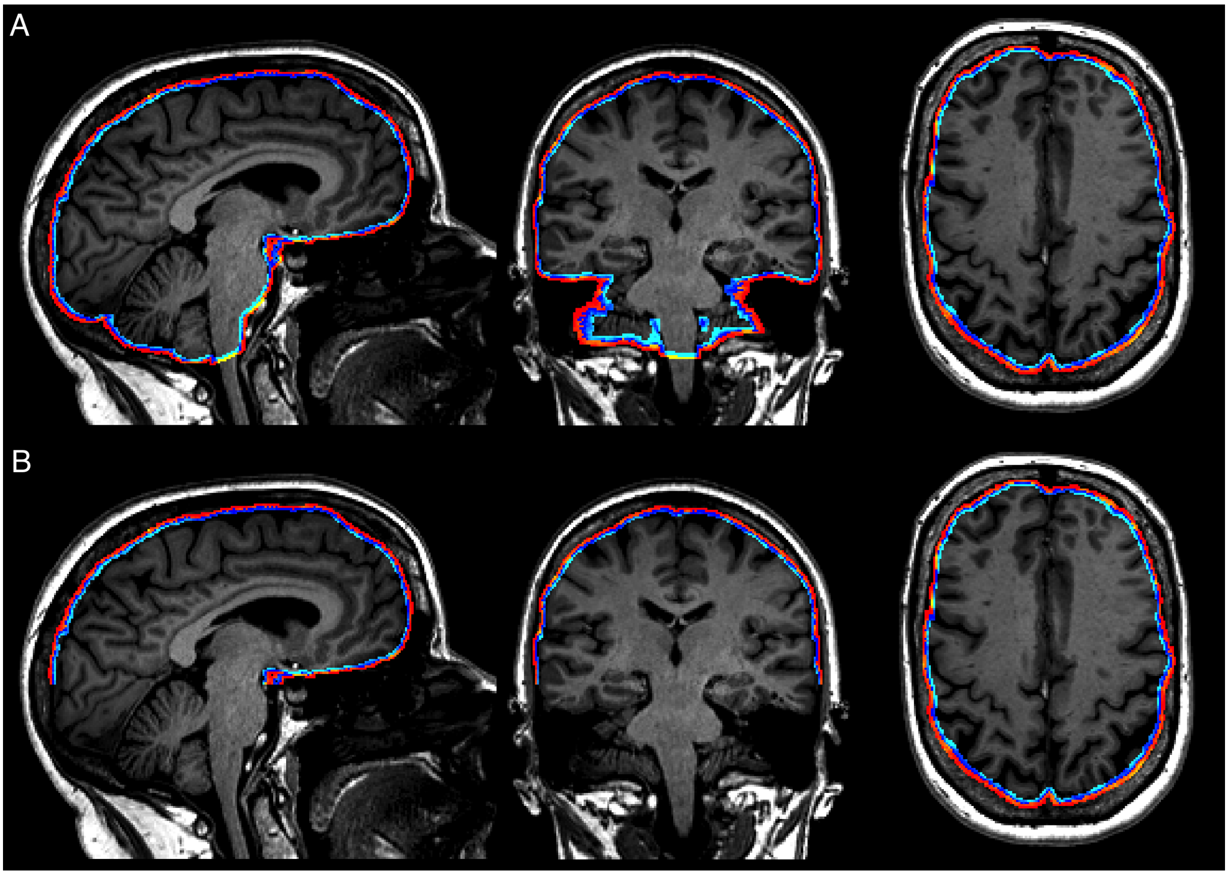

Figure S4: Interior (Blue) and exterior (Red) strip boundary of the brain mask. *A:* Whole brain mask. *B:* Upper half brain mask.

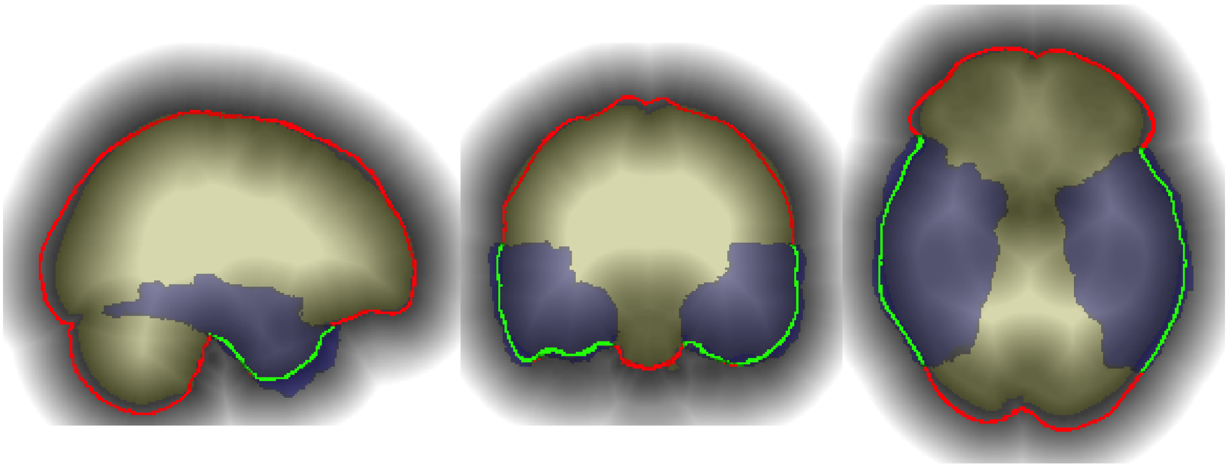

Figure S5: Calculation of the distance between the border of the temporal lobe and the border of the MNI template brain mask. *Greyscale:* Distance map of a voxel to the border of the MNI template brain mask. Higher (brighter) intensities mean greater distance. *Yellow:* Outline of the MNI template brain mask. The previous distance map has 0s in the voxels in the border of this mask. *Red:* Outline of the brain mask of the subject taken to MNI space with a linear (12 DOF) registration calculated between the T1 and the MNI T1 template. *Blue:* Dilated mask of the temporal lobes of the subject (Calculated by applying the inverse non-linear registration from T1 to MNI described in Section 2.2.2 of the paper to a mask of the temporal lobe defined in MNI space) taken to MNI space using the same linear registration mentioned before. *Green:* Intersection between blue and red. Distance map defined in greyscale will be masked with this ROI to calculate a set of statistics.

- The next feature (QC_T1_160) contains the volume of white matter hyper-intensities in structural images using T1 and T2 FLAIR as inputs to BIANCA (Griffanti et al., 2016). This feature may be used to detect atypical structures that could affect registration.
- The next thirty features (QC_T1_161 to QC_T1_190) contain descriptive statistics (Minimum, maximum, median, and 75th and 98th percentile) of the distance between the boundaries of 6 different structures (cerebellum, parietal lobe, temporal lobe, occipital lobe, frontal lobe and total brain), and the boundary of the MNI152 brain mask. Figure S5 shows an example with temporal lobe. These features may be useful in identifying atypical deformations due to poor registration to MNI152 space.

S5. Fieldmap magnitude generation

Running topup returns several outputs like the phase of the estimated fieldmap, the off-resonance field used to correct the geometric distortions, and the corrected input b=0 pair of images. Usually, one of those b=0 images (or the average) is taken as the magnitude fieldmap image (used for registering the dMRI data to structural space), but that may cause some problems in our data due to the tight FOV. These magnitude images will only have non-zero values for voxels where data exists for both images of the pair. Therefore, in the presence of subject movement between the images, there can be zero-voxels introduced into the data.

Therefore, to get a correct fieldmap magnitude, the following steps are needed in both AP and PA (see Figure S6):

- Apply topup warp to the original b=0 image.
- Generate a mask with the zero-valued voxels from the corrected b=0 image from previous step.
- Multiply each of those masks by the corrected b=0 image of the opposite direction and add the result to the corrected b=0 image. That way, for each zero-valued voxel in the corrected b=0 image, we get the corresponding value from the corrected opposite b=0 image.
- Average these.

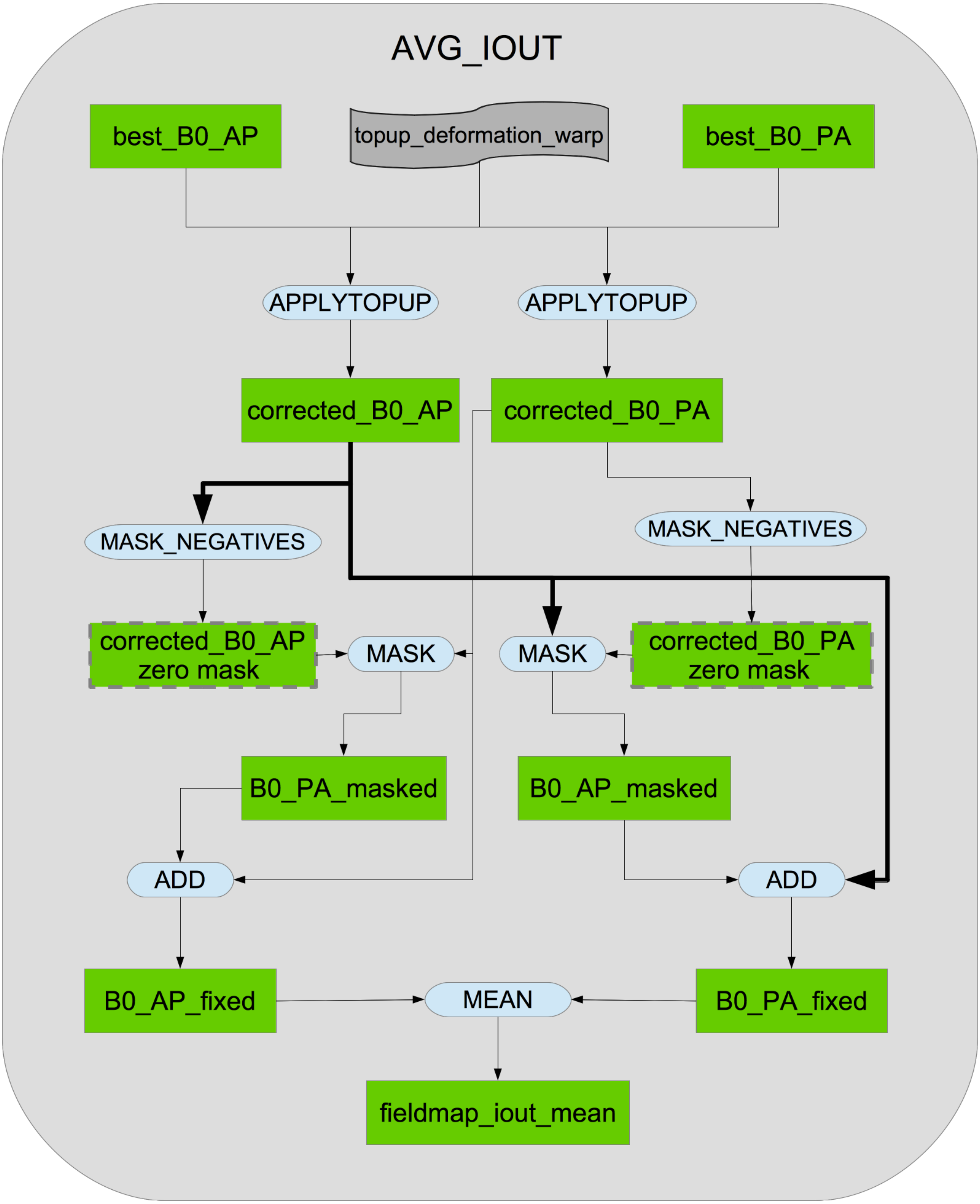

Figure S6: Flowchart for the averaging of the B0s for the fieldmap generation.

S6. Size of the training dataset for QC

The classifier accuracy to detect problematic datasets will naturally improve with increasing training set size (as the classifier will have more information to model the patterns that describe the classes). In order to obtain a somewhat quantitative proof of this idea, we have re-run the classifier accuracy estimation by separating a small subset of the original 5822 subjects (1000) and training the classifier with a progressively smaller fraction of the other 4822 datasets (4770, 4721, etc, until just having 48 in the last iteration).

As shown in Figure S7, a reduction in the size of the training set marginally improves the amount of incorrectly classified datasets in the test set (and also reduces the amount of datasets to manually review). Nevertheless, this small reduction comes at a cost: An increase in the problematic datasets that are missed by the classifier. The explanation for this behaviour is that the less bad subjects to learn from in the training set, the less likely it is to classify a bad subject correctly. Therefore, it is more likely that most of the subjects will be classified as "good". As the proportion of bad:good is very small, even classifying all subjects as good (which is what the classifier is doing in practice with the smallest training set sizes tested here) will have a very good accuracy, although the QC tool is then of course not functioning usefully.

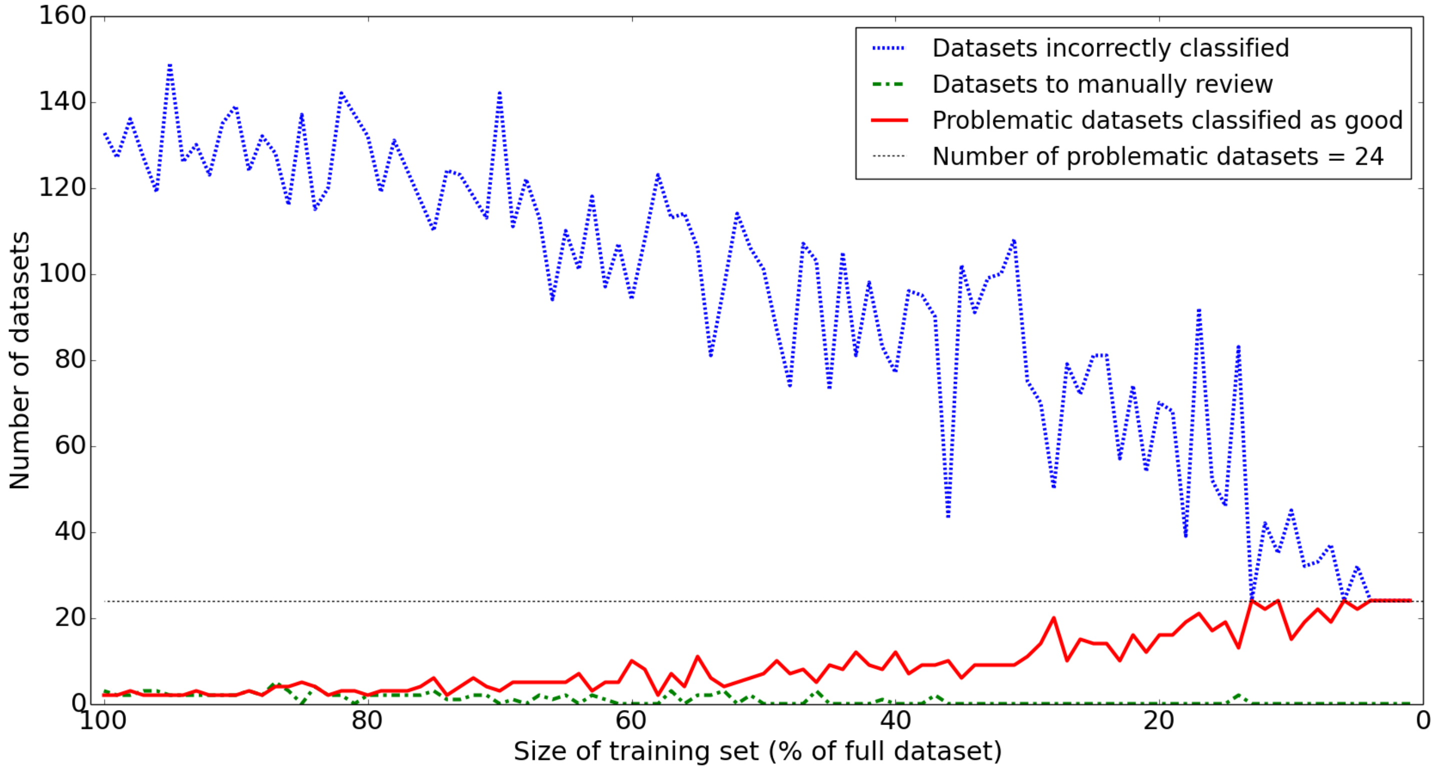

Figure S7: Increase in False Negatives when training size decreases.

S7. Additional Figures and Tables

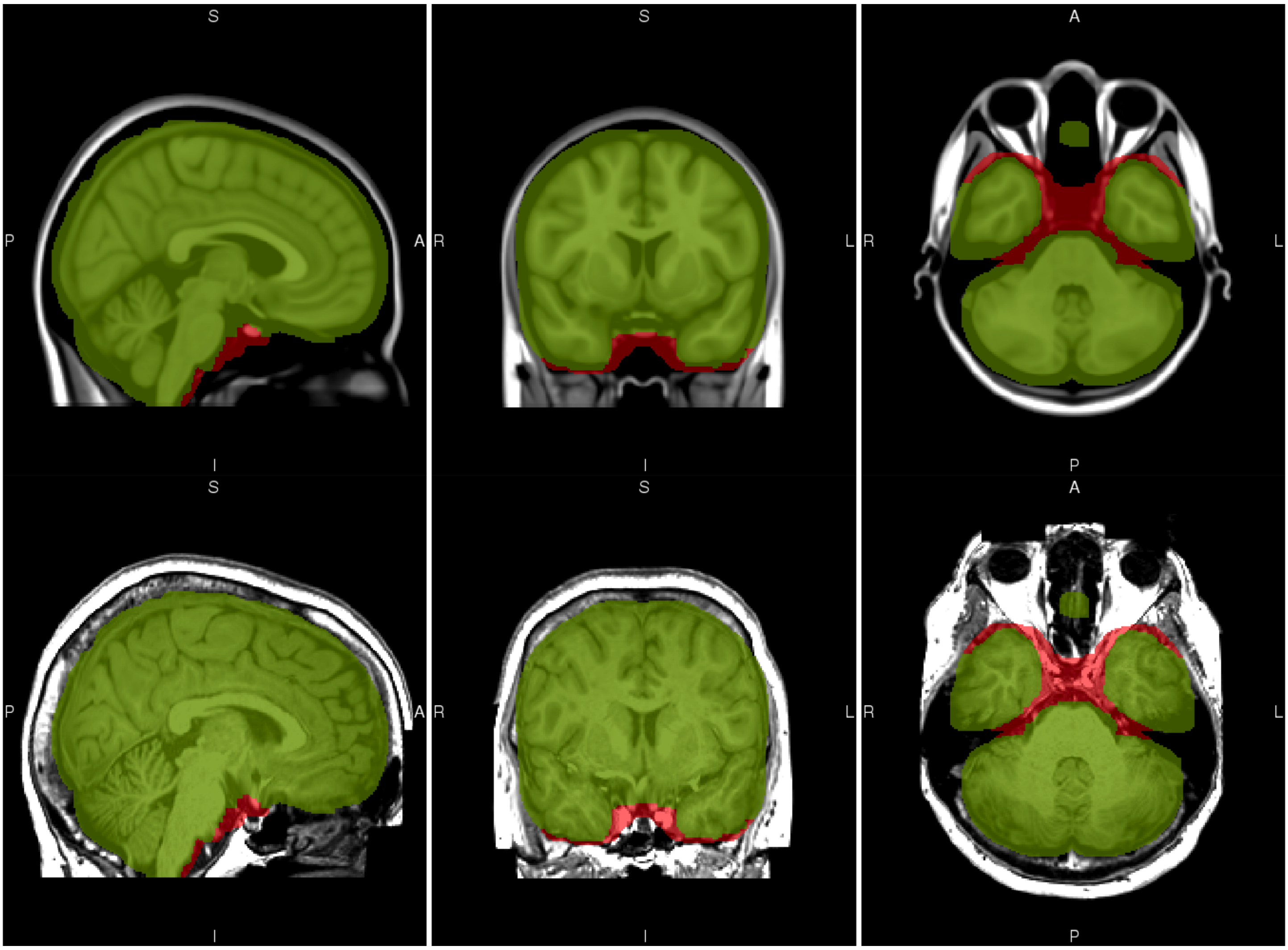

Figure S8: The modified dilated brain mask used to improve non-linear registration. *Top:* MNI template with the two brain masks. The red mask is the original brain mask. The green mask is the modified version for UK Biobank. *Bottom:* Subject registered to the MNI template with the two brain masks. The red mask is the original brain mask. The green mask is the modified version for UK Biobank.

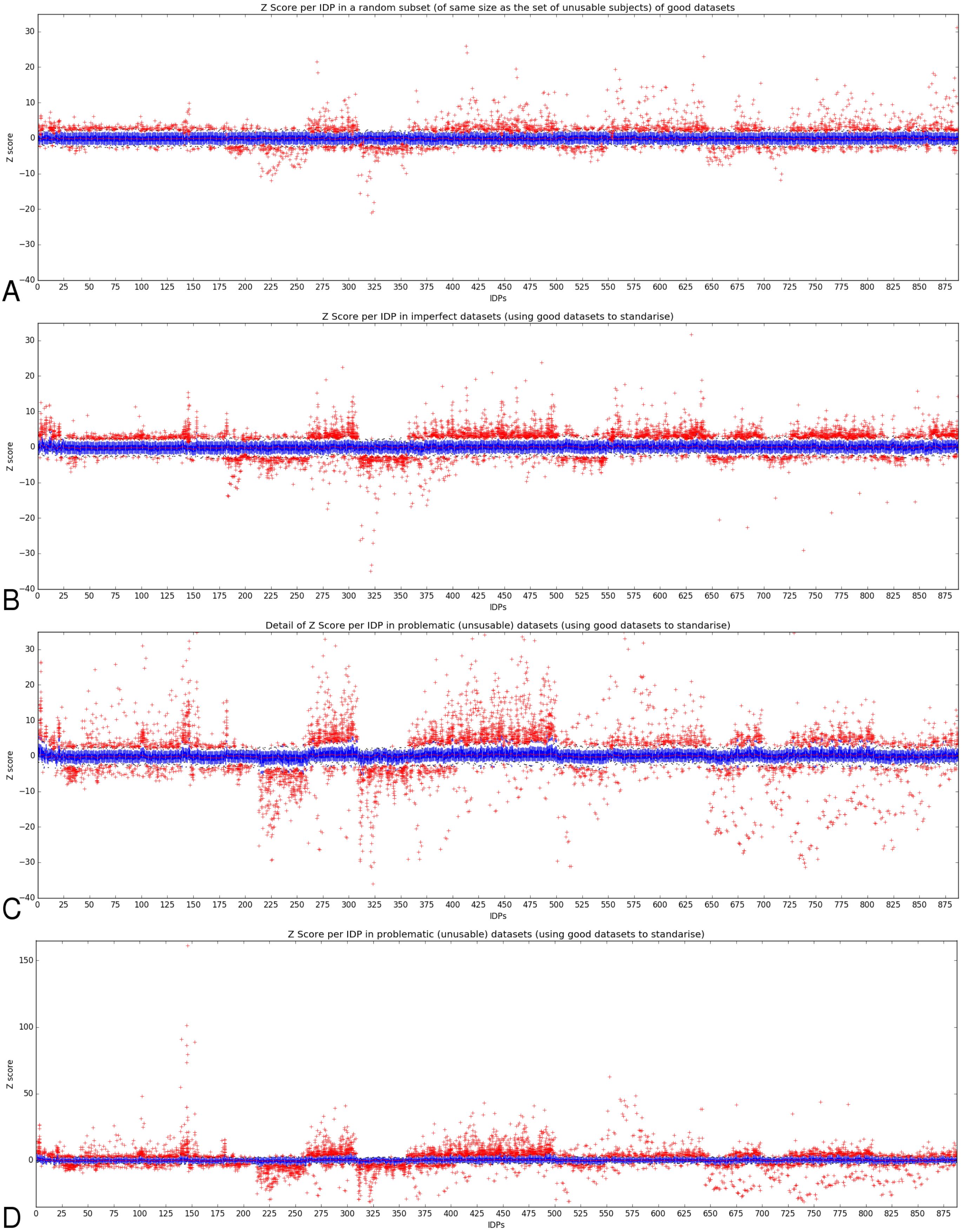

Figure S9: Boxplots with the Z scores for the 888 not-rfMRI IDPs (i.e. excluding netmats, and listed in the same order as described above) for *A:* Good datasets, *B:* Imperfect datasets, *C* and *D:* Unusable ("problem") datasets. Y axis has the same range for A, B and C to ease comparison. Full range for unusable datasets is shown in D. Blue boxes show the IQRs per IDP. Red points above and below are the boxplot-defined outliers.

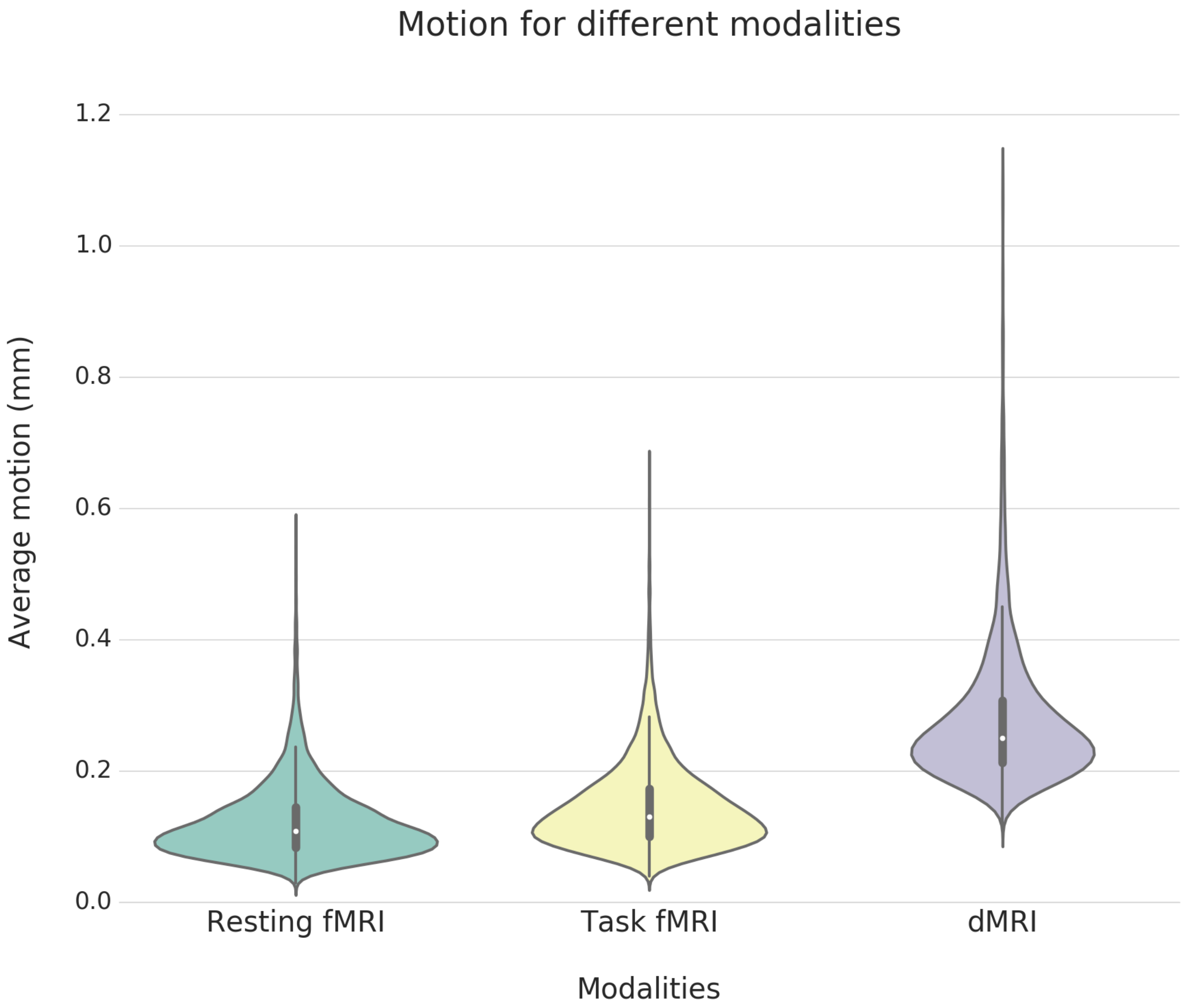

Figure S10: Motion estimation for the temporal modalities. The motion per modality is calculated using the motion of each timepoint relative to the previous one, and averaging the motion displacement across the brain and then across timepoints. For dMRI, motion is calculated without the translation in the AP encoding direction (Y), as that component is highly affected by eddy distortions. Movement is higher in dMRI because, assuming that much of the true movement is slow over time in comparison to the TR, volume-to-volume movement should be approximately proportional to TR, which is 4 times larger. Extreme outliers (points further than: $mean \pm (8 * \sigma)$) have been removed for ease of visualisation."

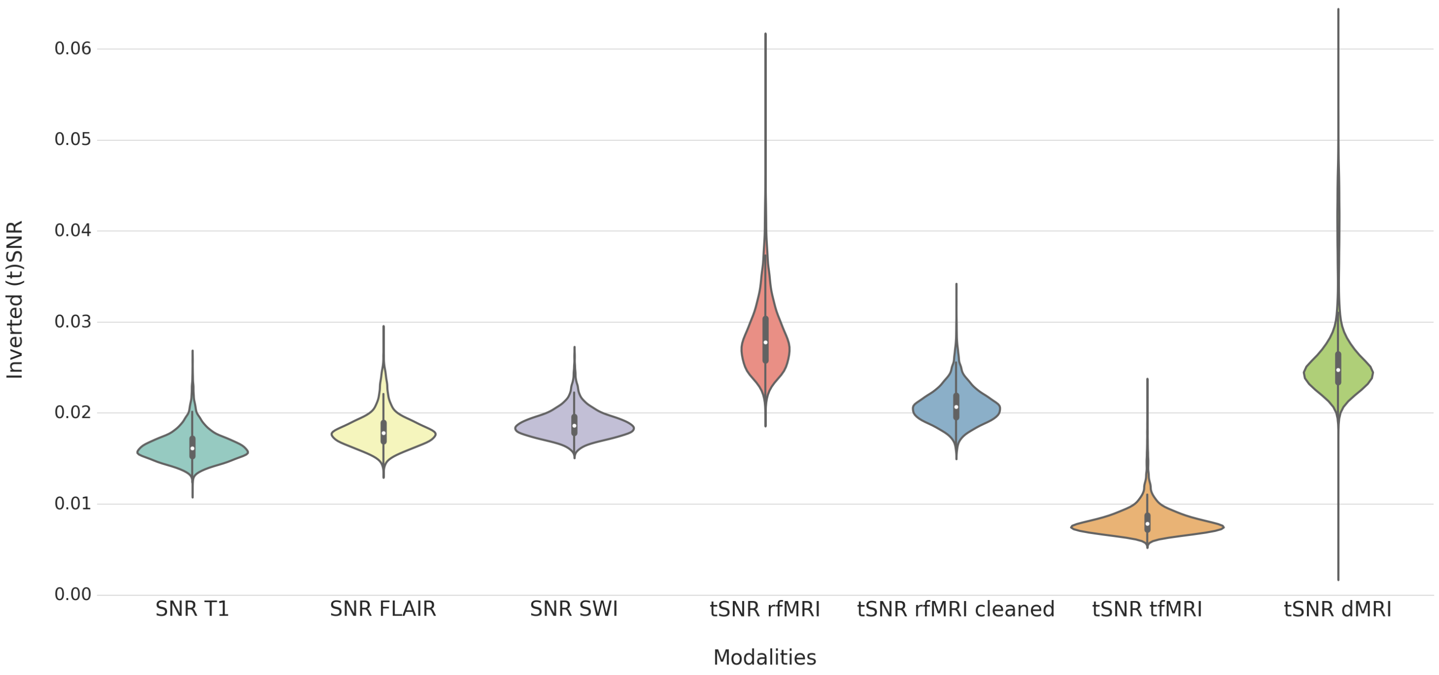

Figure S11: Inverted SNR for T1, T2 FLAIR and SWI (magnitude, first echo) and inverted tSNR for Resting fMRI, Resting fMRI (after removing noise components using FIX), Task fMRI (processing includes spatial smoothing, as explained in section 2.2.8 of the main text) and dMRI (calculated using all b=0 images in the AP direction). Extreme outliers (points further than: $mean \pm (8 * \sigma)$) have been removed for ease of visualisation.

Table S1: Cost matrix for MultiCost classifier

	**0**	**1**	**2**	**3**	**4**	**5**	**6**	**7**
**0**	0	15000	18000	15000	15000	15000	18000	15000
**1**	30000	0	1	1	1	1	1	1
**2**	5000000	1	0	1	1	1	1	1
**3**	30000	1	1	0	1	1	1	1
**4**	30000	1	1	1	0	1	1	1
**5**	3000000	1	1	1	1	0	1	1
**6**	2000000	1	1	1	1	1	0	1
**7**	5000000	1	1	1	1	1	1	0

Cost matrix represents the hypothetical cost of misclassifying a subject belonging to the category X (columns) in the category Y (rows).

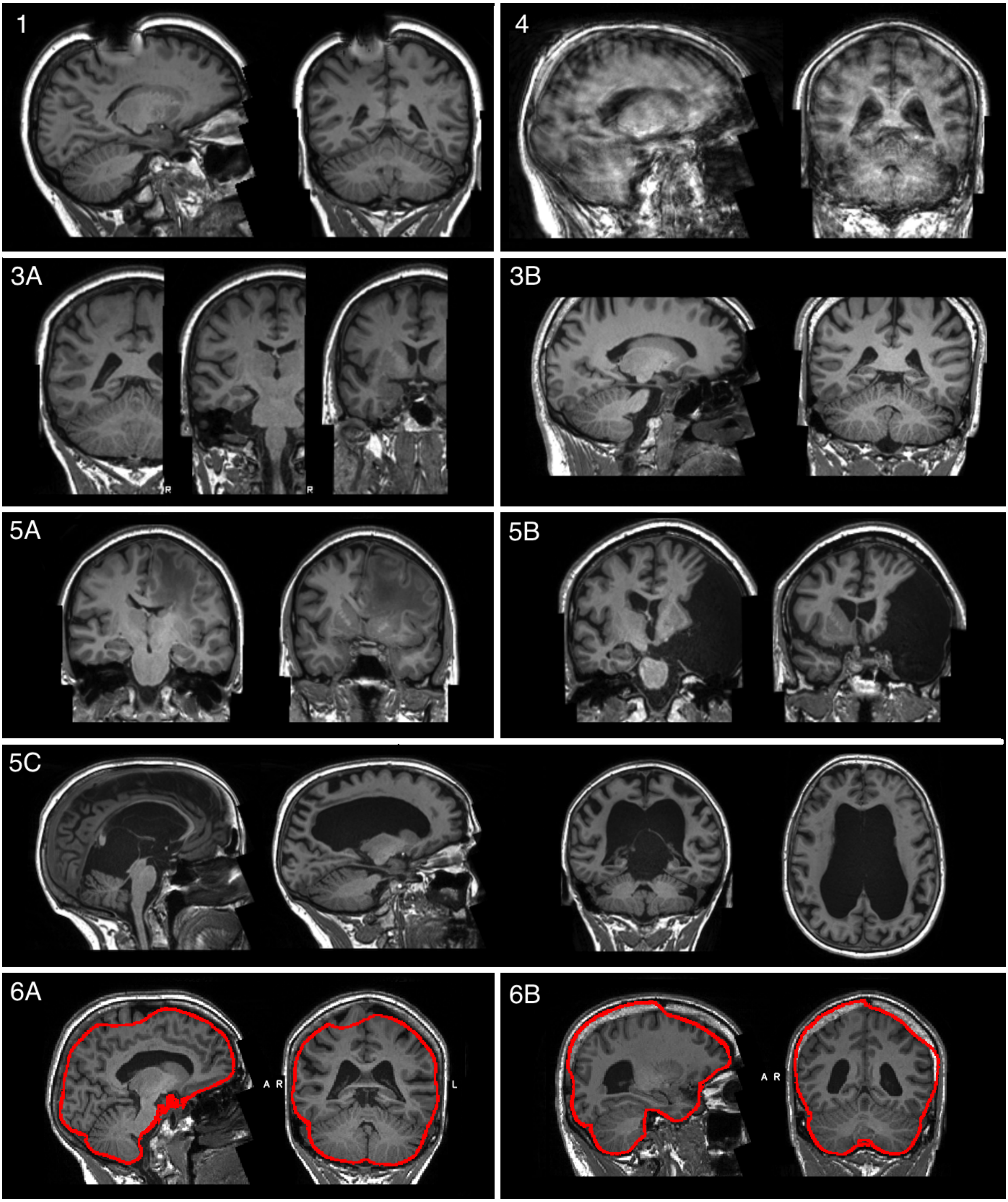

Figure S12: Examples of problematic ("unusable") datasets. Numbers correspond to the classes described in Table 3. *Class 1:* Multiple or Unknown problems. *Class 4:* Bad head motion. *Class 3 (A and B):* Bad FoV. *Class 5 (A, B and C):* Structurally atypical. *Class 6 (A and B):* Bad registration: Unknown reason

Table S2: Classification of problems / imperfections per modality in UK Biobank

Modality	Problem code	Imperfection code
T1	4 = Bad reg: Bad head motion / Noise<br>5 = Bad reg: Structurally atypical<br>6 = Bad reg: Large Ventricles<br>7 = Bad reg: General Registration failure<br>8 = Bad reg: Bad brain mask on the top<br>9 = Bad reg: Bad brain mask on the temporal lobe<br>10 = Bad reg: Brain mask out of the brain	1 = Multiple / Unknown imperfections<br>2 = Bad head movement<br>3 = Movement-related ringing / blurring<br>4 = Bias field / contrast problem<br>5 = Structurally atypical<br>6 = Problem on top (brain mask)<br>7 = Problem on temp. lobe (brain mask)
T2 FLAIR	4 = T1 unusable<br>5 = Bad registration to T1<br>6 = Incompatible (Phase 2)	1 = Multiple / Unknown imperfections
swMRI	4 = T1 unusable<br>5 = Bad registration to T1<br>6 = Distortion<br>7 = Acquisition problem<br>8 = Rotated sform / qform	1 = Multiple / Unknown imperfections
dMRI	4 = T1 unusable<br>5 = Bad EPI distortions<br>6 = Bad bias field<br>7 = Bad registration to T1 or MNI<br>8 = Incompatible (Phase 2)	1 = Multiple / Unknown imperfections
rfMRI	4 = T1 or fieldmap unusable<br>5 = Bad EPI distortions<br>6 = Bad bias field<br>7 = Bad registration to T1<br>8 = Bad acquisition (Movement)	1 = Multiple / Unknown imperfections<br>2 = Missing/corrupt SBRef
tfMRI	(Same as for rfMRI)	(Same as for rfMRI)

Problem codes for all modalities:

- 0 = No problem
- 1 = Multiple / Unknown problems
- 2 = Missing or incomplete modality
- 3 = Bad FoV

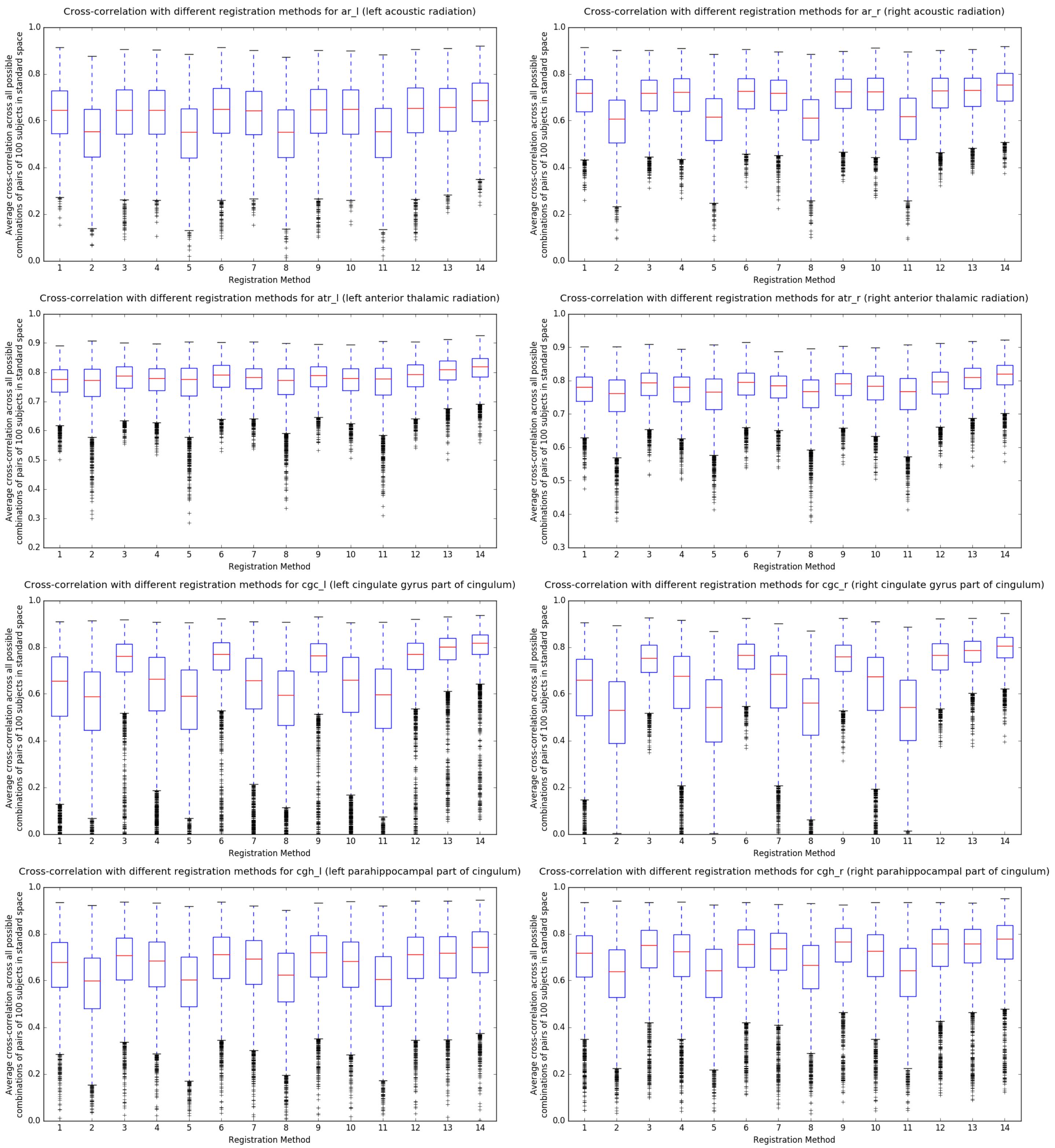

Figure S13: Comparison of 14 different alignment methods of FA to MNI space applied to separate tracts. Data, methodology and registration methods were as used for Fig 13 of the paper. We show here the differences per tract.

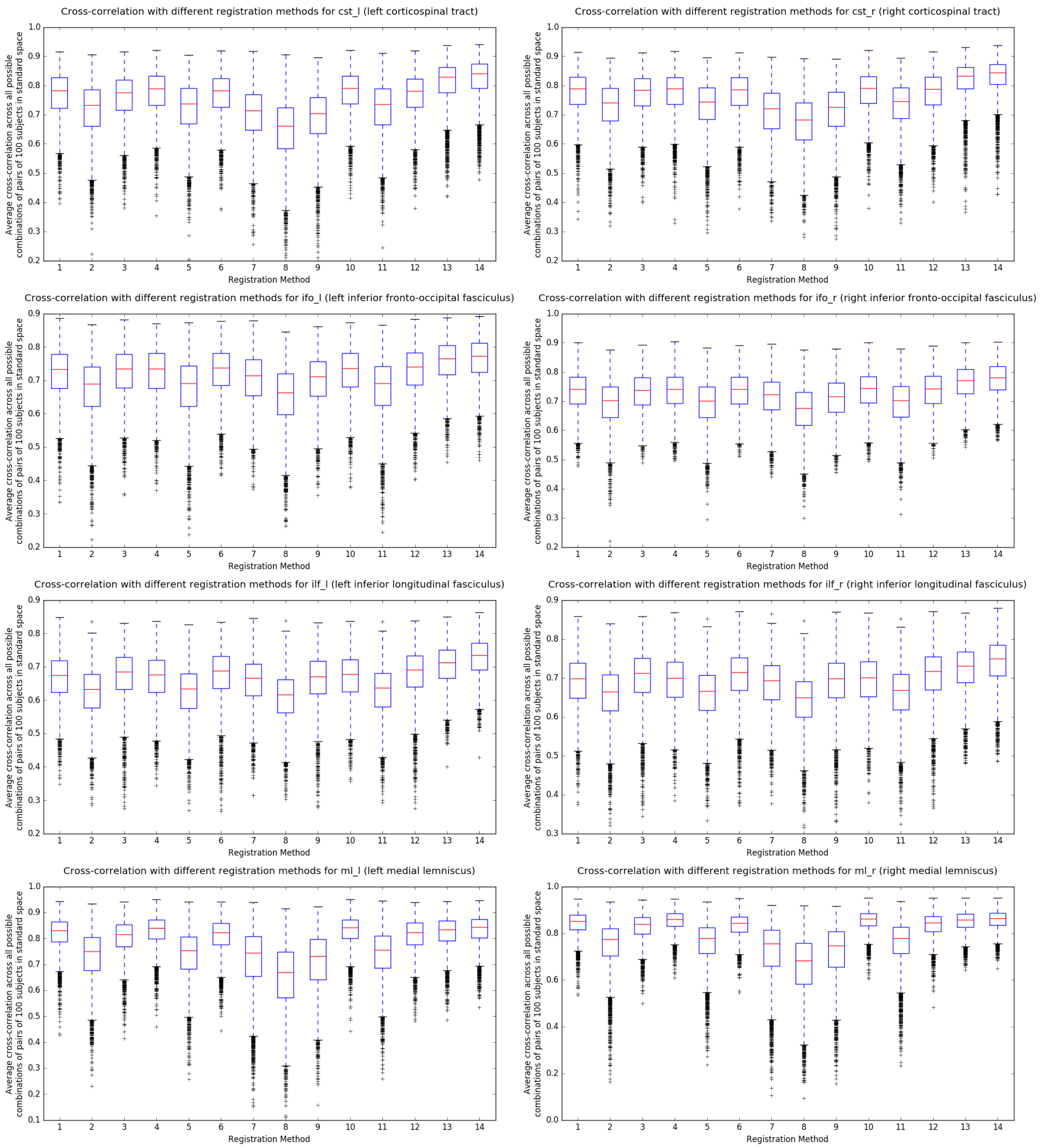

Figure S14: Comparison of 14 different alignment methods of FA to MNI space applied to separate tracts. Data, methodology and registration methods were as used for Fig 13 of the paper. We show here the differences per tract.

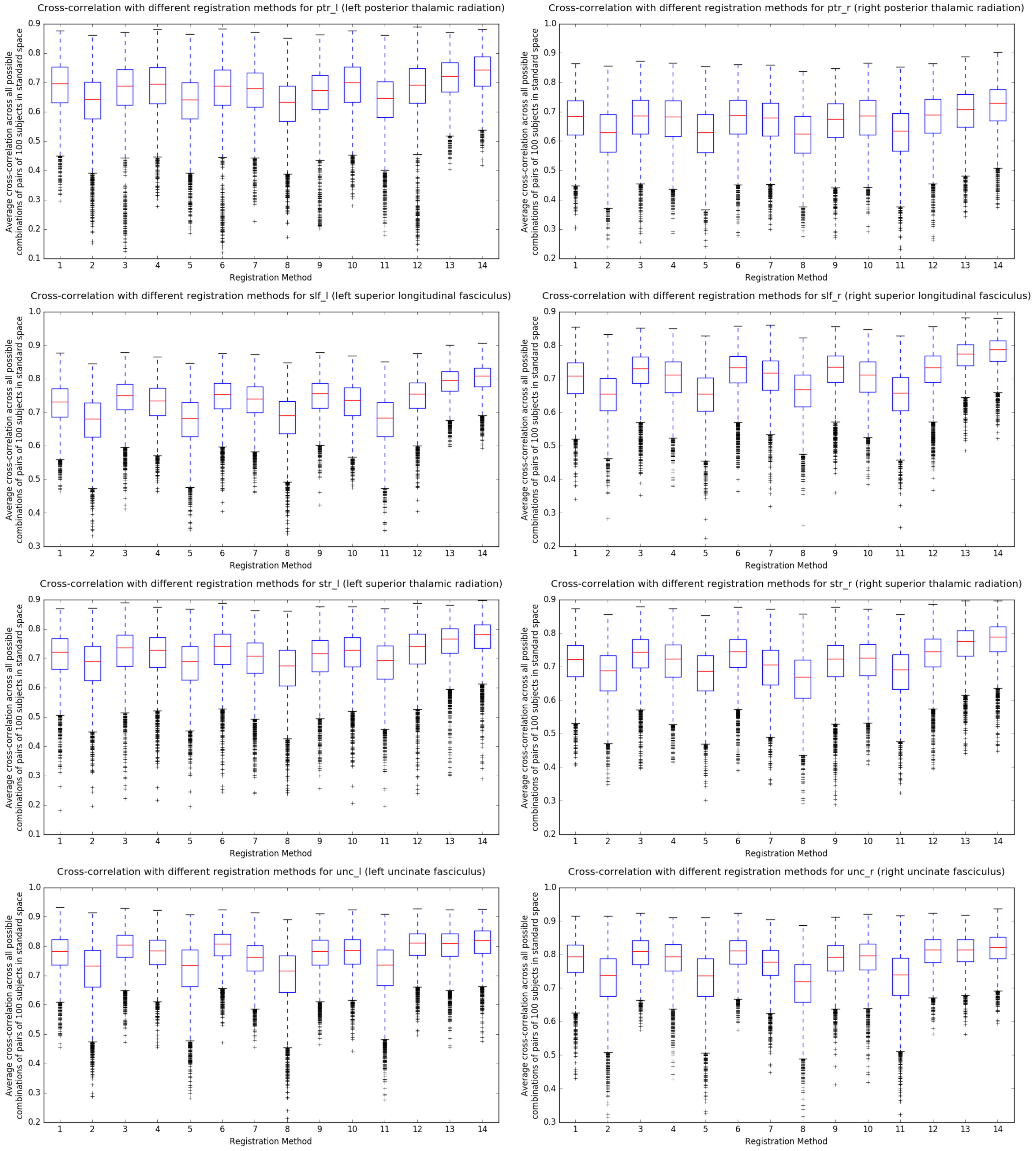

Figure S15: Comparison of 14 different alignment methods of FA to MNI space applied to separate tracts. Data, methodology and registration methods were as used for Fig 13 of the paper. We show here the differences per tract.

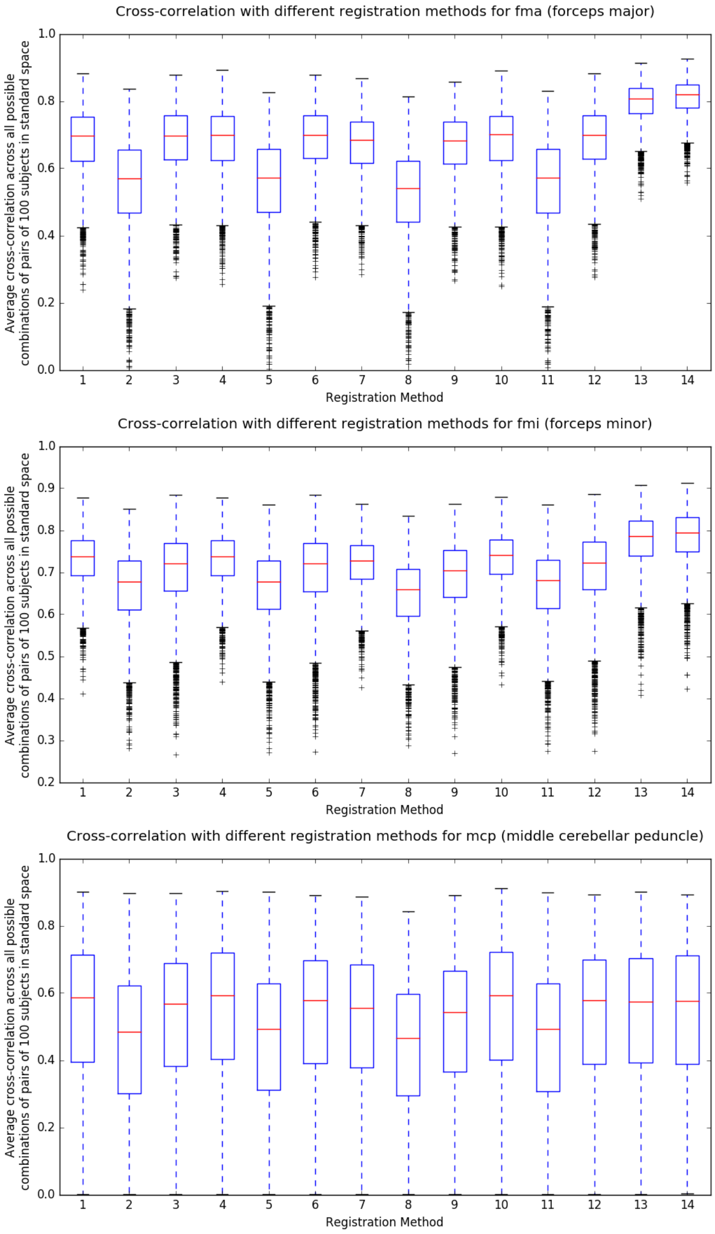

Figure S16: Comparison of 14 different alignment methods of FA to MNI space applied to separate tracts. Data, methodology and registration methods were as used for Fig 13 of the paper. We show here the differences per tract.

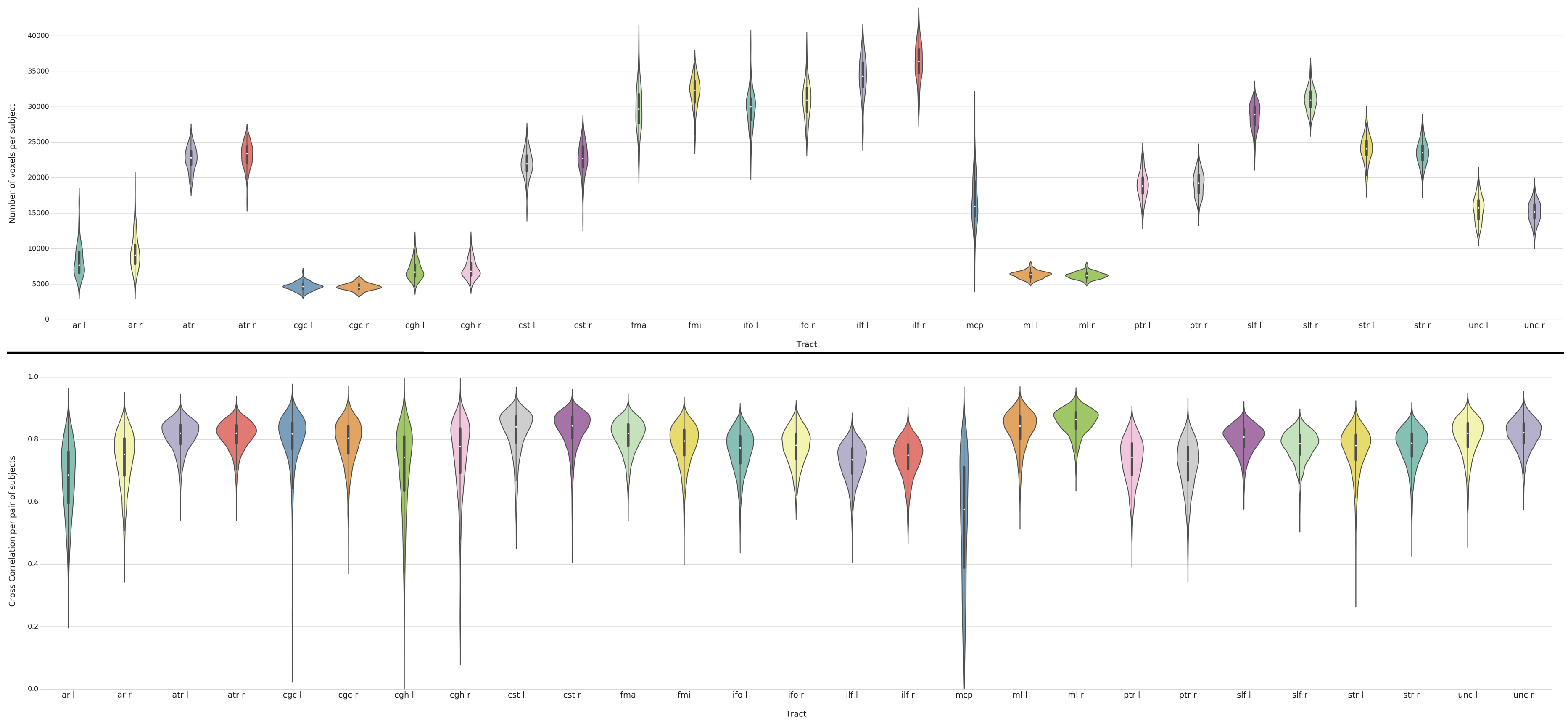

Figure S17: *Top:* Distribution of the number of voxels per tract in MNI space for the 100 subjects after the registration described in Section 3.6.2 of the paper (14th method). *Bottom:* Cross correlation of each tract for all the possible pairs of 100 subjects after registering them to MNI space (again, 14th method). We aim to show how the cross-correlation is not affected by the size of the tract. The meaning of the tract names can be found in https://fsl.fmrib.ox.ac.uk/fsl/fslwiki/AutoPtx

S8. BIBLIOGRAPHY

References

Ashburner, J., Friston, K. J., 2005. Unified segmentation. Neuroimage 26 (3), 839–851.

Griffanti, L., Zamboni, G., Khan, A., Li, L., Bonifacio, G., Sundaresan, V., Schulz, U. G., Kuker, W., Battaglini, M., Rothwell, P. M., et al., 2016. BIANCA (Brain Intensity AbNormality Classification Algorithm): A new tool for automated segmentation of white matter hyperintensities. NeuroImage 141, 191–205.

Iglesias, J. E., Liu, C.-Y., Thompson, P. M., Tu, Z., 2011. Robust brain extraction across datasets and comparison with publicly available methods. IEEE transactions on medical imaging 30 (9), 1617–1634.

Kazemi, K., Noorizadeh, N., 2014. Quantitative comparison of spm, fsl, and brainsuite for brain mr image segmentation. Journal of Biomedical Physics and Engineering 4 (1 Mar).

Ségonne, F., Dale, A., Busa, E., Glessner, M., Salat, D., Hahn, H., Fischl, B., 2004. A hybrid approach to the skull stripping problem in mri. Neuroimage 22 (3), 1060–1075.

Shattuck, D. W., Leahy, R. M., 2002. Brainsuite: an automated cortical surface identification tool. Medical image analysis 6 (2), 129–142.

Smith, S., November 2002. Fast Robust Automated Brain Extraction. Human Brain Mapping 17 (3), 143–155.
